# Precision medicine in inflammatory bowel disease: concept, progress and challenges

**DOI:** 10.12688/f1000research.20928.1

**Published:** 2020-01-28

**Authors:** Simon P. Borg-Bartolo, Ray Kiran Boyapati, Jack Satsangi, Rahul Kalla

**Affiliations:** 1 Department of Gastroenterology, Wythenshawe Hospital, Manchester University NHS Foundation Trust, Southmoor Road, Wythenshawe, Manchester, M23 9LT, UK; 2 Department of Gastroenterology, Monash Health, Clayton, Victoria, Australia; 3 Faculty of Medicine, Nursing & Health Sciences, Monash University, Clayton, Victoria, Australia; 4 Translational Gastroenterology Unit, Nuffield Department of Medicine, Experimental Medicine Division, University of Oxford, John Radcliffe Hospital, Oxford, UK; 5 Department of Gastroenterology, Royal Infirmary of Edinburgh, 51 Little France Crescent, Edinburgh, EH16 4SA, UK

**Keywords:** inflammatory bowel disease, precision medicine, biomarkers, genomics, prognosis, therapeutics, Crohn's disease, ulcerative colitis, microbiome

## Abstract

Crohn’s disease and ulcerative colitis are increasingly prevalent, relapsing and remitting inflammatory bowel diseases (IBDs) with variable disease courses and complications. Their aetiology remains unclear but current evidence shows an increasingly complex pathophysiology broadly centring on the genome, exposome, microbiome and immunome. Our increased understanding of disease pathogenesis is providing an ever-expanding arsenal of therapeutic options, but these can be expensive and patients can lose response or never respond to certain therapies. Therefore, there is now a growing need to personalise therapies on the basis of the underlying disease biology and a desire to shift our approach from “reactive” management driven by disease complications to “proactive” care with an aim to prevent disease sequelae. Precision medicine is the tailoring of medical treatment to the individual patient, encompassing a multitude of data-driven (and multi-omic) approaches to foster accurate clinical decision-making. In IBD, precision medicine would have significant benefits, enabling timely therapy that is both effective and appropriate for the individual. In this review, we summarise some of the key areas of progress towards precision medicine, including predicting disease susceptibility and its course, personalising therapies in IBD and monitoring response to therapy. We also highlight some of the challenges to be overcome in order to deliver this approach.

## Core tip

Our current understanding of inflammatory bowel disease (IBD), whilst incomplete, suggests an increasingly complex pathophysiology. Increasing biologic and small-molecule therapeutic options are available, but loss of response is common. Precision medicine in IBD, with the aim of tailoring the right therapy to the right patient at the right time on the basis of an individual patient’s biology, is an aspiration. We summarise recent progress in the pursuit of precision medicine in IBD and highlight some of the challenges that remain. In the future, precision medicine in IBD has the potential to enable delivery of truly individualised IBD care.

## Introduction

Crohn’s disease (CD) and ulcerative colitis (UC) are forms of idiopathic IBD that follow a relapsing and remitting course ^1, 2^. The incidence and prevalence of IBD are increasing worldwide, particularly in newly industrialised countries ^3– 5^. The pathogenesis of IBD remains unclear, although it is increasingly evident that the pathophysiological factors leading to heterogenous IBD phenotypes are increasingly complex and interwoven ^6^. Broadly, it appears that aetiological factors concentrate around the genome ^7^, exposome ^8^, microbiome ^9^ and immunome ^10^. Whilst many factors have been identified by studying each of these disciplines in isolation, a true understanding of an individual patient’s aetiological and pathogenic mechanisms, and thus therapeutic options, will arise only from an integrated approach ^11^.

A growing understanding of the immunopathophysiological mechanisms underlying the development of IBD has led to significant advances in the ability to treat IBD effectively ^12– 15^. In a relatively short time, we have progressed from “conventional treatment” using corticosteroids with or without immunomodulators to having an increasing array of biologic and small-molecule therapies in our medical arsenal ^16^. However, the associated costs of treating IBD are also increasing ^17– 20^. It is also becoming clear that the first biologic therapy (that is, the initial biologic therapy commenced in an individual patient) seems to be the most effective with a “dampened” response rate using second-line biologics ^21^. Although IBD clinicians embrace increasing therapeutic options for our patients, we also have a responsibility to ensure optimal and efficient use for each individual patient. The ability to predict response, relapse and side effects as well as optimise drug levels and early identification of loss of response is highly desirable ^22^. The development of biomarkers to enable targeted and effective individualised treatment is therefore necessary to allow selection of the right drug at the right time in the right patient ^23^.

So it is clear that a change of perspective is required to advance our understanding of IBD. IBD is a stochastic, heterogenous disease with complex aetiology and increasingly complex treatment options. Traditional research approaches focusing on isolated aspects of IBD aetiology or treatment, whilst necessary and highly informative, run the risk of oversimplification without an appreciation of complex aetiological and pharmacological associations ^11^. There is a growing need for a “multi-omic” approach where data from different disciplines regarding the same subject are integrated to advance our understanding of IBD immunopathogenesis, to identify new drug targets and to identify biomarkers to enable better detection and treatment of disease ^24^. By understanding the complex pathogenesis of IBD, we will be better able to practice precision medicine in which we can tailor medical therapy to the individual patient ^25, 26^.

## Pathogenesis of inflammatory bowel disease

The exact pathogenesis of IBD remains enigmatic. Whilst it is understood that complex interactions between host and environment are pivotal, it is not yet clear exactly how these lead to the various disease phenotypes. It is beyond the scope of this article to provide an in-depth review of IBD pathogenesis, but we will summarise some of the key points.

### Genetics

The observation that the risk of developing IBD is greater in people who have relatives with IBD suggests a genetic component ^27– 29^. Genome-wide association studies (GWASs) have facilitated the discovery of a large number of significant genetic risk loci, some of which are discussed later in this article ^6, 30^. Many loci are shared between UC and CD ^31^ and may overlap with loci associated with other inflammatory diseases ^32^. Functional gene polymorphisms are known to impact on innate immunity, adaptive immunity and regulation of the intestinal barrier ^33^. Despite the advances in genetic understanding, only about 25% of IBD heritability can currently be explained ^30^.

### Environment

The incidence and prevalence of IBD are highest in the Western world, but newly industrialised countries are also experiencing increases in the incidence and prevalence of IBD, and migrants to the Western world acquire the same risk of IBD as that of the native population ^34^. This occurs independently of ethnicity and seems to mirror the industrialisation and westernisation of populations ^4^. Early life events, pollution, and diet have all been implicated in the development of IBD ^8^.

### Microbiome

The dysbiosis seen in IBD has been well described as being characterised by a reduced microbial diversity, expansion of facultative anaerobes and decreased numbers of obligate anaerobes ^35^. In the most comprehensive analysis carried out to date on the microbiome in IBD, longitudinal profiles of 132 subjects were developed, demonstrating disruptions in microbial transcription and metabolite pools as well as increases in temporal variability associated with disease activity ^36^. It remains unknown which of these observed changes are the cause or consequence of IBD and this remains an area of intense investigation.

### Immunome

The gut is a crucial immunological interface that maintains immunological balance by appropriately recognising and tolerating commensal bacteria, food antigens and self-antigens (tolerogenic response) whilst identifying and acting against pathogenic organisms (immunogenic response) ^6^. Dysregulated immunological responses within the gut leading to imbalances in pro- and anti-inflammatory pathways involved in innate and adaptive immunity are thought to be pivotal in the development and persistence of inflammation in IBD ^37^. Impaired barrier function of the epithelium coupled with epithelial neutrophil accumulation, defective antigen clearance by macrophages and impaired conditioning of dendritic cells are all innate immune factors thought to contribute to the persistence of inflammation ^10^. Dendritic cells bridge the gap between innate and adaptive immunity by inducing tolerance or immunogenicity amongst T and B cells and therefore can be influential in mucosal homeostasis versus mucosal inflammation ^38^. The balance between effector T cells (T _H_1, T _H_2, T _H_17 and T _H_9) and regulatory T (T _reg_) cells is crucial to maintaining tolerance or promoting chronic inflammation ^39^.

## Concept of precision medicine and its relevance to inflammatory bowel disease

Precision medicine refers to the “tailoring of medical treatment to the individual characteristics of each patient” ^40^. Whilst the concepts of personalised medicine and precision medicine are very similar, precision medicine also encompasses a multidisciplinary data-driven approach to foster better clinical decision-making through a clear understanding of the molecular basis of an individual’s disease ^41^. In 2015, the national Precision Medicine Initiative, a novel and ambitious plan to collect multi-omic data on over 1 million patients in a new national cohort in an attempt to accelerate understanding of diseases and their treatments, was announced in the US ^42^. This high-profile endorsement of precision medicine has served to ignite interest in and raise awareness of the concept.

Precision medicine in IBD is conceptually attractive. We know that patients with CD or UC can have a markedly variable disease course. In Norwegian cohorts followed up over 10 years, the IBSEN study group found that up to 53% of patients with CD developed stricturing or penetrating disease and that up to 19% of patients with UC required colectomy by 10 years ^43, 44^. Traditional “step-up” therapy risks undertreating those patients who are destined to develop complications, but “top-down” therapy risks overtreating patients who may have remained stable and complication-free on less expensive therapies and exposes those patients to unnecessary side effects. Many clinical parameters, such as serological markers, disease location, disease behaviour, age and lifestyle, have been found to be associated with disease severity ^45, 46^. However, none is reliable or specific enough to alter early IBD management. Therefore, the ability to predict aggressive disease behaviour, high risk of disease complications, or response to certain treatments at or near the time of diagnosis for individual patients currently remains elusive. Nonetheless, there are encouraging signs of progress. This review aims to summarise some of the key progress made to date in the pursuit of precision medicine in IBD and aims to outline some of the challenges that have yet to be overcome.

## Predicting disease susceptibility and clinical phenotype

GWASs have provided important insights into the aetiology and development of IBD ^30^. To date, over 240 IBD susceptibility loci have been identified through this approach ^47– 50^. Some of the key biological processes uncovered by GWASs include epithelial barrier dysfunction, disruption of antimicrobial defences and immune dysregulation. Whilst GWASs have provided numerous avenues to explore, these have yet to filter through as clinically useful biomarkers to help predict disease and its phenotype.

Some GWAS associations have been associated with distinct clinical phenotypes. *NOD2/CARD15* is involved in pattern recognition receptor signalling in response to microbial stimuli and has been associated with an ileal fibrostenosing disease phenotype ^51– 54^. *NOD2/CARD15* has also been associated with the need for surgery and complicated disease course ^55^. *ATG16L1*, which implicates defective autophagy as an important part of CD development ^56^, has been associated with ileal disease ^57^, and another autophagy gene, *IGRM*, has been associated with penetrating disease ^58^. *IL23R*, which has become a successful therapeutic target in CD ^59, 60^, has also been previously linked to ileal CD ^61^. However, most of these individual genes have limited effect sizes, meaning that applicability on their own to the general population is limited. Recent insights into the genetics of IBD reveal distinct clinical phenotypes. Exploring the associations between genetic risk scores and IBD sub-phenotypes in 29,838 patients, the UK IBD Genetics Consortium (IBDGC) was able to redefine disease subtypes into ileal CD, colonic CD and UC ^62^.

Similarly, the microbiome may have a role to play in helping identify “high risk” individuals. In a study of new-onset IBD, Gevers *et al*. found ileal microbiome signatures to be predictive of CD, even in the absence of overt inflammation ^63^. It is likely that the microbial signature of IBD will vary depending on the clinical phenotype ^64^ as well as environmental factors such as smoking and medication use. Longitudinal studies have suggested an ability to predict CD and UC phenotypes on the basis of the microbiome and an ability to distinguish patients with IBD from those with similar symptoms ^65^. In the future, there is the enticing possibility of generating “risk scores” for patients on the basis of their microbiome ^66^.

Despite this tremendous progress in genetics and the microbiome, screening for IBD in the clinic continues to rely on a combination of clinical symptoms and blood-based biomarkers. More recently, faecal calprotectin (FC) has emerged as a robust screening tool for those with suspected IBD. Studies have shown that FC has a pooled sensitivity and specificity of 0.93 (0.85–0.97) and 0.96 (0.79–0.99) respectively and a high negative predictive value (NPV) (0.96–0.98) ^67, 68^. FC now represents one of the core tests in clinical practice in the UK (and elsewhere) as a screening tool to identify those who require further tertiary-care investigations for suspected IBD ^69^. Despite its value, several practical issues reported in the literature appear to hinder its widespread clinical uptake. First, patient compliance with FC is often poor. A recent study showed that only one third of patients were compliant with FC testing, forgetfulness being the main reason for non-compliance ^70^. Two recent studies investigating the acceptability of FC in IBD found that sample collection was a major barrier to testing; blood testing was preferred over stool testing ^71, 72^. These “patient factors” pose a real challenge for clinicians in implementing FC testing in clinical care and hamper its clinical utility. FC also shows within-day variations, and the ideal time of sample collection is unclear ^73, 74^.

Researchers are beginning to explore novel blood-based diagnostic markers across multiple -omics platforms, including epigenetics, metabolomics and proteomics, and the results have been promising. Multicentre consortia such as IBD-Character, IBD-BIOM and Biocycle have been funded by the European Commission to develop novel biomarkers that can be transitioned into the clinic; each focuses on specific unmet needs in clinical practice. Whereas IBD-Character and IBD-BIOM have focussed on developing diagnostic and prognostic multi-omic markers in IBD, Biocycle explores innovative regimes for maintenance treatment in moderate to severe CD ^75^. These have yet to be translated to clinically useful biomarkers but offer a glimpse into the potential for an expanded repertoire to help diagnose IBD wherever it is suspected. However, multiple challenges exist: biomarkers should have key qualities such as being simple, easy to perform, minimally invasive, rapid and reproducible, and should consistently show high accuracy in predicting disease across several populations ^76^. These characteristics have yet to be ascertained amongst the biomarkers studied so far.

## Predicting disease course

Historically, gastroenterologists have relied on the established clinical predictors of disease course ( Table 1) to help tailor management. Over time, we have progressed from relying on clinical predictors to using biomarkers to predict outcomes and tailor treatment algorithms based on disease course. Markers such as FC have been shown to be associated with the need for colectomy in UC ^77^. Similarly, in a recent retrospective study, FC was shown to predict progression in CD behaviour, hospitalisation for a flare and surgery ^78^. There are, however, conflicting reports on its prognostic utility ^79^. Furthermore, the reported prognostic performance of FC is similar to that of other non-specific blood markers such as C-reactive protein (CRP). It is clear that, despite the available predictors, accurate prognostication at the time of IBD diagnosis has yet to be achieved.

**Table 1.** Clinical parameters that predict unfavourable inflammatory bowel disease course.

Disease	Time frame	Predictor of unfavourable course	References
Crohn’s disease	Within 5 years of diagnosis	Age < 40	88
		Need for steroids in first flare	88, 89
		Perianal disease	88, 89
		Upper gastrointestinal lesions	90
		Ileocolonic lesions	91
	Within 10 years of diagnosis	Age < 40	44, 92
		Upper gastrointestinal lesions	92
		Stricturing and penetrating behaviour	44
		Terminal ileal lesions	44
Ulcerative colitis	Within 5 years of diagnosis	Younger age	90
		Female gender	90
	Within 10 years of diagnosis	Younger age	93
		Female gender	93
		Fewer systemic symptoms	94
		Extensive colitis	43, 94, 95
		Non-smoking status	93

We are, however, beginning to unearth the molecular profiles that associate with disease progression in IBD. The seminal study by Lee *et al*. ^80^, who explored the CD8 T-cell transcriptomic profiles in newly diagnosed IBD, identified unique RNA signatures that were associated with the need for treatment escalation or surgery (or both) over time in both CD and UC. In their follow-on study, Biasci *et al*. developed a multi-gene signature predicting the need for escalation using original criteria in UC (hazard ratio [HR] 3.1, 95% confidence interval [CI] 1.25–7.72, *P* = 0.02) and CD (HR 2.7, 95% CI 1.32–5.34, *P* = 0.01) ^81^, although this profile differs from the original T-cell profile signature. Using the same criteria for escalation, the UK IBDGC identified four prognostic genetic loci: *FOXO3, XACT*, a region upstream of *IGFBP1* and the MHC region ^82^. These genes were distinct from those that predict CD susceptibility. The molecular architecture of disease course has been further defined beyond genetics at a methylome, glycome and proteome level. Studies have shown that patients with an aggressive disease course display unique circulating methylome and proteome signatures ^83– 85^, including markers such as serum calprotectin ^79^, that predict treatment escalation or surgery (or both) over time. Glycomic markers have previously been shown to be associated with IBD ^86^ and more recently have shown the ability to predict treatment escalation ^87^. All of these studies have similar clinical criteria for escalation, based on step-up approach treatment algorithms. In clinical practice, tailoring early “top-down” therapies in those with disease progression while avoiding potent therapies in those with a benign disease course at diagnosis is a real unmet need. It has yet to be ascertained whether this approach will improve clinical outcomes.

Other equally relevant definitions of disease course are being studied by IBD consortia across populations. One such consortium is the Risk Stratification and Identification of Immunogenetic and Microbial Markers of Rapid Disease Progression in Children with Crohn’s Disease (RISK) study ^96^. Defining aggressive disease course as a progression in CD behaviour to either penetrating or stricturing complications over time, this prospective inception cohort study identified unique multi-omic profiles that associate with disease progression. Ileal transcriptomic data showed that expression of inflammatory response to microbe signatures versus extracellular matrix upregulation signatures discriminated between later-penetrating versus stricturing complication development. The addition of ileal transcriptomic data to a clinical and serologically based competing-risk score improved the sensitivity and specificity of the score ^96^. The RISK study group has also shown that by integrating summary-level GWAS and expression quantitative trait loci with RNA-seq data, transcriptional risk scores can be generated which outperform genetic risk scores in identifying CD and are able to predict CD disease course over time ^97^. Randomised controlled trials (RCTs) are needed to determine whether early characterisation and therapy based on these profiles have the ability to alter disease course over time in paediatric CD.

Microbial populations may have a role in helping predict disease course, as illustrated by a study in post-operative recurrence in CD ^98^. Here, the authors demonstrated that a decreased population of *Faecalibacterium prausnitzii* in the resected ileum correlated with a higher rate of recurrence ^98^. In a study of paediatric CD, gut microbial signatures at the time of diagnosis were found to help predict 6-month steroid-free remission ^99^. These studies demonstrate conceptually the potential for microbiome signatures to provide clinicians with prognostic information to help inform treatment decisions, although longitudinal studies and further validation are required.

Understanding the progression of IBD at diagnosis using several distinct yet clinically important criteria at a multi-omic level will help personalise treatment algorithms based on biology rather than symptomatology with an aim to improve clinical outcomes over time. Empowering patients with this information at diagnosis may aid progress towards personalising care in IBD.

## Personalising therapies in inflammatory bowel disease

The array of treatment options in IBD has grown dramatically over recent years, and a large number of therapies are in the pipeline ^13^. Ultimately, the goal of precision medicine is to enable preferential selection of a specific therapy based on an individual patient’s biology whilst individualising dosing to ensure that therapeutic effects are maintained and side effect risk minimised. In current clinical practice, use of therapeutic drug monitoring (TDM) “biomarkers” is well established in order to increase the chances of response and reduce risk of side effects. Despite this, it is clear that currently available TDM strategies do not completely mitigate against the risk of adverse events. Large multicentre trials such as CALM have reported an adverse event rate of up to 24 to 26% using current anti-tumour necrosis factor (anti-TNF) therapies ^100^.

In the case of thiopurines, we are able to predict the risk of life-threatening myelosuppression by measurement of thiopurine methyltransferase (TPMT) enabling dose reduction with intermediate and low levels at the point of treatment commencement ^101^. Until recently, NUDT15 polymorphisms were known to be associated with the onset of thiopurine-induced myelosuppression and were thought to be most relevant in Asian populations ^102, 103^. However, a recent multicentre, case control study of patients of European ancestry either affected or unaffected by thiopurine-induced myelosuppression has altered this perception ^104^. In that study, a GWAS and exome-wide association study (EWAS) approach identified an association between a NUDT15 variant and myelosuppression ^104^. Carriage of any three coding NUDT15 variants was associated with an increased risk of myelosuppression. The number needed to genotype was 95, at par with TPMT ^104^. These data suggest that there may be a role for NUDT15 sequencing in addition to TPMT sequencing prior to commencing thiopurines. HLA type has also been demonstrated to be a predictor of side effects in thiopurine therapy. In a multicentre study, 433 patients who had developed thiopurine-induced pancreatitis within 3 months of starting thiopurine therapy and case controls were identified. Using a GWAS approach, an association with pancreatitis development was found at rs2647047 which was found to link to HLA-DQA1 and HLA-DRB1 alleles. Compared with a baseline risk of about 4% for thiopurine-induced pancreatitis in all patients, those heterozygous for rs2647047 had a 9% risk and those who were homozygous had a 17% risk ^105^. During thiopurine therapy, thiopurine metabolites can be measured to determine whether a particular drug dose is optimised to produce a beneficial therapeutic effect whilst identifying poor compliance where it exists and minimising risks of side effects in an individual patient ^106, 107^.

Similarly, TDM for biologic therapies is increasingly embedded in clinical practice. Anti-TNF therapy monitoring with measurement of trough drug levels and detection of anti-drug antibodies allows optimisation of therapeutic effect and directs decisions to switch biologic therapy in the case of primary and secondary non-response ^108^. In their RCT, Steenholdt *et al*. compared reactive TDM for anti-TNF therapy with empiric dose escalation in the setting of secondary loss of response ^109^. In that RCT, TDM was associated with significant cost reductions. In the TAXIT ^110^ and Tailorix ^111^ RCTs, anti-TNF dosing based on proactive TDM was compared with dosing based on clinical features. In the TAXIT trial, there was a significantly reduced relapse rate and modest cost saving in the proactive TDM group. By contrast, the Tailorix trial found that there was no significant benefit of proactive TDM over-dosing based on clinical features. Overall, anti-TNF TDM is associated with reduced cost and improved durability of response, but there is no conclusive evidence that a proactive or reactive approach is superior ^112^.

Accurate pre-treatment prediction of response to biologic therapy would enable better drug selection for patients. Currently, there are no clinically available biomarkers that accurately predict treatment response, although a large number of potential candidates have been studied and extensively reviewed recently ^26^. Here, we report a number of new and noteworthy examples. West *et al*. demonstrated reproducibly in a number of data sets that high expression of oncostatin M (OSM) (a cytokine) is associated with reduced anti-TNF response ^113^. Verstockt *et al*. showed that low serum expression of Triggering Receptor Expressed on Myeloid cells 1 (TREM1) measured prior to commencing therapy is an accurate anti-TNF–specific marker for future anti-TNF response with endoscopic remission in both CD and UC ^114^. Telesco *et al*. identified and validated a 13-gene signature that predicted week 6 mucosal healing response (area under the curve receiver operating characteristics (AUC _ROC_) = 0.688, *P* = 0.002) to golimumab in the PURSUIT and PROgECT studies. The low specificity of this 13-gene signature may impact on the utility of this multi-gene panel ^115^. In a novel approach, Morilla *et al*. identified and validated a nine-microRNA signature in colonic biopsies in acute severe UC, successfully classifying responders and non-responders to corticosteroids, infliximab and cyclosporine in 90%, 84% and 80% of patients respectively ^116^.

Prediction of response to biologic therapy using the microbial signatures of patients with IBD has also been a focus of recent investigation. Faecal microbial diversity resembled controls in paediatric patients who responded to anti-TNF therapy compared with non-responders ^117^. Similarly, Ananthakrishnan *et al*. found differences in the distal gut microbiome of CD patients who responded to anti-integrin therapy compared with non-responders ^118^. The authors reported higher abundance of *Roseburia inulinivorans* and a *Burkholderiales* species as well as enrichment of 13 microbial pathways in those achieving remission. Furthermore, a neural network algorithm was able to predict drug response, although, interestingly, these findings were not apparent in UC. For ustekinumab, using 16S rRNA gene sequencing, Doherty *et al*. reported that at week 6 into therapy, patients in remission could be distinguished from those with active disease by characterisation of their microbiome ^119^. Although these studies collectively show promise as proof of concept, large-scale validation is required before these tools can become part of clinical practice.

Predicting treatment failure is equally important to potentially facilitate early switching of therapy and increase the likelihood and cost-effectiveness of recapturing response. Recently, results from the Personalised Anti-TNF Therapy in Crohn’s Disease Study (PANTS) were published ^120^. This multicentre, prospective, observational cohort study included 1610 patients from the point of first anti-TNF therapy exposure and followed them up for 12 months or until withdrawal of anti-TNF therapy. Low drug concentrations at week 14 were associated with primary non-response and non-remission at week 54. Obesity at baseline was associated with non-remission at week 54 for adalimumab only. Immunogenicity by week 54 was seen more commonly in patients on infliximab than on adalimumab (62.8% versus 28.5% respectively). Drug concentrations at week 14 were an independent risk factor for immunogenicity for both drugs. Obesity in the case of adalimumab and smoking in the case of infliximab were also associated with immunogenicity. Immunomodulator use was the main protective factor, and thiopurine-related reduction in immunogenicity occurred in a dose-dependent fashion. The PANTS study group has also presented data showing that HLA variants determine anti-TNF therapy immunogenicity and HLA-DQ1*05 genotype was a key factor ^121^. Attention has also focussed on the role of glycosylation. This complex post-translational modification of proteins affects their function and can be significantly altered in inflammation ^91^. Pereira *et al*. recently demonstrated that lower levels of branched N-glycans in colonic biopsies from a cohort of 131 patients with UC were predictive of failure to respond to standard therapy (5-ASA, corticosteroids and immunomodulators) ^122^. The authors also demonstrated that the sensitivity and specificity of the predictive effect were different at time of diagnosis, within 5 months of diagnosis, and at time of diagnosis in patients presenting with severe disease. The only other independent predictor of failure of standard therapy was demonstrated to be high CRP. When low levels of branched N-glycans and high CRP levels were combined in all UC versus severe UC patients at the time of diagnosis, the probability of failure of standard therapy was 46.6% versus 76.5% respectively, sensitivity was 69% versus 88% respectively, and specificity was 80% versus 75% ^122^.

More recent studies have used single-cell gene expression technologies in order to better understand the molecular pathophysiology of drug response. In active UC, there appears to be a differential reduction in epithelial mitochondrial genes in active UC, including the known master regulator of mitochondria biogenesis *PPARGC1A* ( *PGC1α*) and epithelial mitochondrial membrane potential ( *MMP*) ^123^. Intestinal epithelial depletion of *PGC1α* has been shown to negatively impact on mitochondrial function and subsequent epithelial barrier; barrier dysfunction is a feature of UC pathogenesis ^124^. This study also identified a gene signature that associates with anti-TNF and anti-integrin therapy response ^123^. In CD, a recent study investigating cellular subsets in ileal CD identified a unique cellular module in inflamed tissue that includes IgG plasma cells, mononuclear phagocytes, activated T cells and stromal cells (GIMATS module) ^125^. The presence of this module in patients at diagnosis was associated with a failure to achieve durable corticosteroid remission with anti-TNF therapy ^125^.

## Monitoring response to therapy

Disease monitoring and “treat to target” (that is, using predefined outcome measures, such as clinical or endoscopic remission, as goals for the optimisation of therapy) are increasingly being recognised as a “gold standard” in IBD care. Mucosal healing is now considered a robust target and a clinical end-point for more recent drug trials ^126, 127^. In CD, the Selecting Therapeutic Targets in Inflammatory Bowel Disease (STRIDE) programme set out guidelines for the “treat to target” approach in order to obtain clinical and endoscopic remission ^128^. STRIDE recommended the use of FC and CRP to monitor response; however, until recently, there was no consensus on how to use these markers to adjust therapies. Following this, the CALM trial tested the efficacy of tight control management using 3-monthly biomarkers versus conventional management using Crohn’s Disease Activity Index on clinical and endoscopic outcomes in moderate to severe CD ^100^. Significantly higher numbers of patients achieved mucosal healing in the “tight control” arm compared with the conventional group. Furthermore, follow-up data from this study showed that patients with disease monitoring using biomarkers and a goal of achieving mucosal healing in the first year of treatment were less likely to have disease progression over a median of 3 years ^129^.

Monitoring response to therapy has been studied using conventional biomarkers such as FC and CRP. Heida *et al*., in a systematic analysis of six studies, showed that two consecutive elevated FC predicted disease relapse within the following 2 to 3 months ^130^. However, the study was not able to define an optimal FC cutoff for monitoring. An FC cutoff of not more than 250 μg/g was shown to predict endoscopic remission (positive predictive value 48.5% and NPV 96.6%) ^131^. Similarly, CRP has been shown to correlate with clinical disease activity and endoscopic inflammation. However, CRP is a non-specific inflammatory marker and can often be raised in other clinical scenarios such as infections. Furthermore, some patients with active disease can have normal CRP levels ^132^.

An area of intense interest is how best to monitor response with therapeutic de-escalation ^133^. The most robust study of anti-TNF de-escalation was the STORI trial, in which Louis *et al*. prospectively followed 115 patients with CD on combination therapy who discontinued the anti-TNF after at least 6 months of corticosteroid-free clinical remission ^134^. The investigators found that those who experienced a relapse and those who did not had different evolution of CRP and FC with a marked and sudden rise in these biomarkers 4 months prior to relapse ^135^. Several studies have explored other biomarkers of response and, in particular, mucosal healing. In a study looking at blood transcriptional biomarkers, initial whole blood microarray analysis was performed by using Affymetrix GeneChip technology to identify changes in transcriptional gene expression in endoscopically active UC compared with normal controls ^136^. After quantitative reverse transcription polymerase chain reaction (RT-PCR) validation, transcription of neutrophil-related genes, including *CD177*, *haptoglobin*, *G protein–coupled receptor 84*, and *S100 calcium-binding protein A12*, were demonstrated to be most associated with endoscopic disease activity. The above factors were also shown to decrease in association with a reduction in endoscopic disease activity after 14 weeks of anti-TNF treatment in a further group of patients with UC.

Glycoprotein acetylation (GlycA) is another example of a novel biomarker. In a prospective pilot study of patients with IBD, serum GlycA levels were measured via high-throughput nuclear magnetic resonance spectroscopy to investigate its role as a disease activity biomarker. GlycA was shown to normalise to healthy control levels in patients achieving mucosal healing and to significantly decrease in patients who had evidence of improvements in endoscopic appearances regardless of the treatment used. GlycA was also shown to be a useful marker of response in patients with normal CRP levels at baseline. When compared with FC and CRP, GlycA was the only marker to show consistent significant differences between responders and non-responders regardless of the treatment given. GlycA may have advantages over CRP as it is a composite marker originating from a number of acute-phase proteins and so is more stable than CRP. The cost of GlycA measurement was reported to be comparable to that of FC ^137^.

A number of studies have also investigated the applicability of faecal immunochemical testing (FIT) to assess disease activity and mucosal healing in UC. In a recent systematic review, the pooled sensitivity and specificity of FIT to predict mucosal healing were found to be 0.77 (95% CI 0.72–0.81) and 0.81 (95% CI 0.76–0.85) respectively, which were similar to previously published sensitivities and specificities of FC in predicting mucosal healing ^138^. In a study comparing sequential FIT and FC measurements in 84 UC patients having more than one colonoscopy over a 33-month period, the accuracy of assessment of disease activity and mucosal healing was studied ^139^. Where the previous colonoscopy and faecal markers had previously demonstrated mucosal healing, there was increased accuracy of FIT versus FC in predicting mucosal healing at the next colonoscopy. Where the previous colonoscopy and faecal markers had previously shown active inflammation, there was again increased accuracy of FIT versus FC in predicting mucosal healing at the next colonoscopy. However, where there was inflammation seen on both colonoscopies, FC better reflected changes in endoscopic activity than FIT. This interesting study suggests that FIT and FC provide slightly different information in disease monitoring in UC and that measuring both may be of benefit.

Given the well-characterised changes in the microbiome of patients with IBD, restoration of the microbial diversity of patients has been explored as a potential biomarker to assess response to therapy. Restoration of gut microbial diversity has been seen in response to anti-TNF therapy ^140^. In a study of paediatric IBD, Shaw *et al*. found that dysbiosis was correlated with inflammatory burden and that in UC, but not in CD, improvements in faecal diversity correlated with clinical response ^141^.

When taken together, the above studies demonstrate the potential utility of traditional and novel biomarkers as surrogates for endoscopic healing and indicators of pre-clinical relapse. Currently, it seems clear that a combination of clinically available biomarkers should be used to monitor disease activity and response to therapy. There is, however, a need for novel specific biomarkers to translate to clinical practice.

## Future challenges and horizon of precision medicine in inflammatory bowel disease

**Figure 1.**
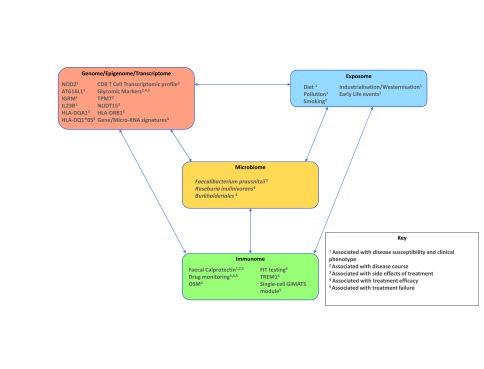
Current progress towards precision medicine in inflammatory bowel disease. This figure summarises some of the progress that has been made towards precision medicine in inflammatory bowel disease and the likely complex inter-relationship of multi-omic data.

**Figure 2.**
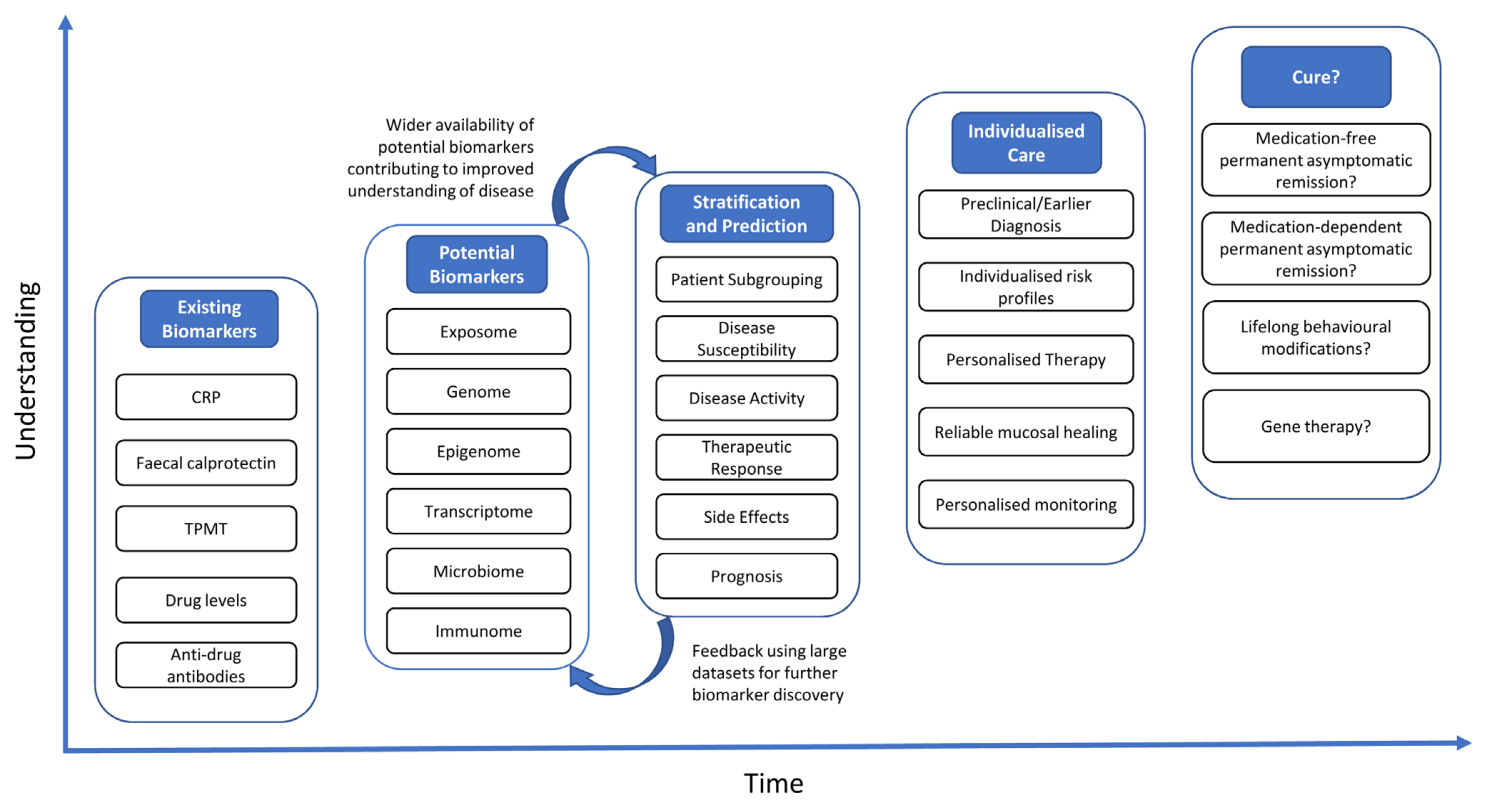
The evolution of precision medicine in inflammatory bowel disease. CRP, C-reactive protein; TPMT, thiopurine methyltransferase.

Substantial progress has been made in our ability to treat CD and UC. With the increasing array of treatment options available, the need for precision medicine in IBD as a whole is paramount. Currently available biomarkers have improved our ability to assess and monitor disease activity and therapeutic efficacy, but each has its own limitations. The aim of precision medicine in IBD is to deliver truly individualised care so that the entire patient journey from diagnosis to treatment is based on the specific biology underlying IBD in the individual patient. The current progress towards precision medicine and likely complex inter-relationships of these multi-omic data in IBD are depicted in Figure 1. Whilst there has been progress, there is still a significant path ahead before precision medicine is achieved in IBD. Our proposed pathway to precision medicine in IBD is shown in Figure 2. As our understanding improves, through using current and novel biomarkers, we will be able to better predict and stratify disease which will positively feedback into an improved ability to identify areas for further biomarker and therapeutic development. This iterative approach will increase our ability to precisely target an individual patient’s inflammation on the basis of their molecular profile, allowing matching of the patient to the most appropriate therapy and monitoring. The overall aim will be to eventually achieve cure rather than simply mucosal or histological healing.

There remain many challenges in order to deliver effective precision medicine in IBD. With the need to look towards big data approaches to make progress in biomarker discovery and improving outcomes in IBD, new approaches to high-quality data sharing are required ^24^. This will require infrastructural changes involving sharing of data between research groups and eventually involving the use of electronic health records. The eventual goal would be to have electronic health records that are able to integrate seamlessly with large clinical and research databases to facilitate early adoption of advances in knowledge whilst providing real-time, up-to-date support to clinicians in making decisions regarding their patients ^142^. There will also need to be new approaches to how data are analysed to maximise their effect. With large amounts of data, there will be an increasing reliance on complex algorithms, including the use of machine learning to make sense of big data coming from multiple sources ^143, 144^. However, there must be stringent checks to ensure that the quality of the data is maintained and that the hypotheses generated are subjected to rigorous validation through pre-clinical and real-world trials prior to adoption in wider medical practice ^24^. One of the biggest barriers to precision medicine in IBD is the lack of a clearly defined mechanism (or mechanisms) of disease ^145^. As we discover new biomarkers and learn new ways of practicing medicine in IBD more precisely, we must use these to interrogate the existing understanding and challenge existing pathophysiological models.

However, there are grounds for significant optimism. A number of ongoing studies will advance our understanding of precision medicine in IBD. The Predicting outcomes For Crohn’s disease using a molecular biomarker (PROFILE) trial (ISRCTN11808228) is the first biomarker-stratified trial in IBD ^146^. It is currently recruiting and aims to recruit 400 patients from about 50 centres across the UK. In that trial, a gene expression signature previously found to determine more aggressive disease ^80^ will be tested for by means of a whole blood quantitative polymerase chain reaction assay that has been previously validated ^147^. Trial participants in each biomarker group will be randomly assigned 1:1 to receive accelerated step-up therapy or top-down therapy. The primary outcome measure for that trial will be sustained surgery and steroid-free remission, and one of the secondary outcome measures will be mucosal healing. The PRognostic effect of Environmental factors in Crohn’s and Colitis (PREdiCCt) Study (ISRCTN67248113) is also currently recruiting and aims to recruit a total of 3100 patients with CD or UC in remission. The study aims to follow these patients over two years and will collect information on diet, lifestyle and gut bacteria to see how these are associated with IBD flare and recovery.

Following the efforts of several consortia to generate multi-omic datasets, there is now a need to define the IBD “interactome”: a disease network where perturbations in individual -omes cause intestinal inflammation, mediated by dysfunctional molecular modules ^11^. There are now several systems biology software tools that facilitate multi-level data integration such as iCluster ^148^ that identifies patient subtypes within IBD on the basis of multi-omic high-throughput data while other tools allow visualisation of the interactome such as Cytoscape ^149^. Methodologies that facilitate data integration are comprehensively summarised in a recent review ^11^. Future drug innovations based primarily on IBD network interactions will transform the field of therapeutics in IBD ^11^.

## Conclusions

Significant progress has been made towards the goal of precision medicine in IBD, but clearly there is still a substantial unmet need for further biomarkers with which to stratify patients and their treatment. It is vital that we continue to develop existing practice by assimilating advances in knowledge whilst optimising novel and existing biomarkers to continually make advances towards truly individualised care. Precision medicine in IBD will require not only the knowledge but the tools and the infrastructure with which to implement it effectively. This will require coordinated investment in technology to enable seamless sharing of big data and the ability to perform high-throughput analysis to create a continually learning IBD community. The recent announcement of the HDRUK G.I. Know Health Data Research Hub for IBD across the UK means that positive steps are already being taken towards integrated big data discovery to advance the field of precision medicine in IBD ( https://www.hdruk.ac.uk/infrastructure/the-hubs/g-i-know/). Precision medicine has huge potential to impact on outcomes in IBD. We all have a responsibility to play our part in practicing high-quality biomarker-driven IBD care whilst contributing to big data in IBD so that in the future we will be able to offer truly individualised IBD care.
